# Increasing prevalence and high incidence of celiac disease in elderly people: A population-based study

**DOI:** 10.1186/1471-230X-9-49

**Published:** 2009-06-29

**Authors:** Anitta Vilppula, Katri Kaukinen, Liisa Luostarinen, Ilkka Krekelä, Heikki Patrikainen, Raisa Valve, Markku Mäki, Pekka Collin

**Affiliations:** 1 Department of Neurology, Päijät-Häme Central Hospital, Lahti, Finland; 2 Department of Gastroenterology and Alimentary Tract Surgery, Tampere University Hospital, Tampere, Finland; 3 Medical School, University of Tampere, Tampere, Finland; 4 Department Internal Medicine, Päijät-Häme Central Hospital, Lahti, Finland; 5 University of Helsinki, Department of Education and Development in Lahti, Helsinki, Finland; 6 Paediatric Research Centre, University of Tampere and Tampere University Hospital, Tampere, Finland

## Abstract

**Background:**

Celiac disease may emerge at any age, but little is known of its appearance in elderly people. We evaluated the prevalence of the condition in individuals over 55 years of age, and determined the incidence of biopsy-proven celiac disease (CDb) and celiac disease including seropositive subjects for anti-tissue transglutaminase antibodies (CDb+s).

**Methods:**

The study based on prevalence figures in 2815 randomly selected subjects who had undergone a clinical examination and serologic screening for celiac disease in 2002. A second screening in the same population was carried out in 2005, comprising now 2216 individuals. Positive tissue transglutaminase antibodies were confirmed with small bowel biopsy.

**Results:**

Within three years the prevalence of CDb increased from 2.13 to 2.34%, and that of CDb+s from 2.45 to 2.70%. Five new cases were found among patients previously seronegative; two had minor abdominal symptoms and three were asymptomatic. The incidence of celiac disease in 2002–2005 was 0.23%, giving an annual incidence of 0.08% in this population.

**Conclusion:**

The prevalence of celiac disease was high in elderly people, but the symptoms were subtle. Repeated screening detected five biopsy-proven cases in three years, indicating that the disorder may develop even in the elderly. Increased alertness to the disorder is therefore warranted.

## Background

Celiac disease is a common disorder affecting more than one percent of the population in the Western world [1]. Serologic screening enables detection of individuals with atypical or subtle symptoms, or even symptomless cases [2]. The condition is often assumed to involve children and young adults. On the contrary, we recently revealed a high number of both diagnosed and undetected celiac disease among elderly people [3]. It remains obscure whether the number of undetected cases in the elderly is due to diagnostic delay, or to the development of celiac disease at an advanced age, or both. The question is important in contemplating whether celiac disease should be actively sought in elderly people, and whether seronegativity could exclude celiac disease once and for all. The aim of this study was to show the current prevalence and incidence of biopsy-proven celiac disease in individuals over 55 years of age. Given the high specificity of serum endomysial (EmA) and tissue transglutaminase antibodies (tTGA) for overt or forthcoming celiac disease, the frequency of seropositivity was likewise investigated.

## Methods

The original study population comprised 4272 randomly selected individuals born in the years 1946–50, 1936–40 and 1926–30; the study sample was representative of the general population in the respective age groups. Altogether 2815 (66%) consented to participate in the original study. Their data were collected for a 10-year research project on Ageing and well-being (Good Ageing in the Lahti region = GOAL) [4]. Sera were collected in 2002, and tested for celiac disease antibodies in 2004. At that time, the number of clinically detected celiac disease cases was evaluated, and new seropositive cases underwent small intestinal biopsy for confirmation of celiac disease. The Amsterdam criteria were applied in the diagnosis of the condition [5]. In the first population screening in 2002 the frequency of diagnosed celiac disease cases was 0.89%, that of screen-detected 1.24% and that of biopsy-proven cases together with cases seropositive without histological confirmation of the disorder 2.45%; these data have been published elsewhere [3].

In 2005, all eligible patients were asked to undergo a new serologic testing. Of the previously tested 2815 patients, 2216 consented. Again, clinically detected celiac disease cases were scrutinized. All sera were tested for IgA class tTGA; positive samples were further tested for IgA class EmA. IgA class tTGA were detected by enzyme-linked immunosorbent assay (Celikey, Phadia, Freiburg, Germany) and the limit of positivity was 5 arbitrary units; IgA class EmA were detected by an indirect immunofluorescence method using human umbilical cord as antigen; a dilution of 1:≥5 was considered positive [6].

All tTGA-positive patients without previous diagnosis of celiac disease were offered upper gastrointestinal endoscopy (irrespective of the EmA titre); four small intestinal biopsies were taken form the distal part of the duodenum and stained with hematoxylin-eosin. The diagnosis of celiac disease was based on typical lesion in small intestinal mucosa.

In the prevalence estimations, subjects with previously detected celiac disease and new biopsy-proven cases found by clinically or screening were included; they are defined in this report as biopsy-proven celiac disease (CDb). The combined prevalence of biopsy-proven and seropositive cases included in addition individuals with positive tTGA but no histological verification of celiac disease (CDb+s).

The incidence of biopsy-proven celiac disease (CDb) was calculated in the 2216 subjects who were tested both in 2002 and 2005, and those seropositive without histological confirmation were added in the combined incidence figures (CDb+s), as defined in the prevalence figures.

### Screening of New Cases

In the original on Ageing and well-being project, there were 199 individuals whose sera were not available in 2002, but consented to screening in 2005. The prevalence of CDb and CDb+s in this group was estimated separately.

The study was accepted by the Ethical committee of Päijät-Häme Central Hospital, and written informed consent was obtained from all participants.

### Statistical Analysis

Prevalence figures were calculated with 95% confidence intervals.

## Results

In the first evaluation, 61 had had been diagnosed with celiac disease (one additional case was found upon re-examination of the case records after the first publication) [3]. All 61 were alive in 2005, and were thus included in the new prevalence data.

**Figure 1:**
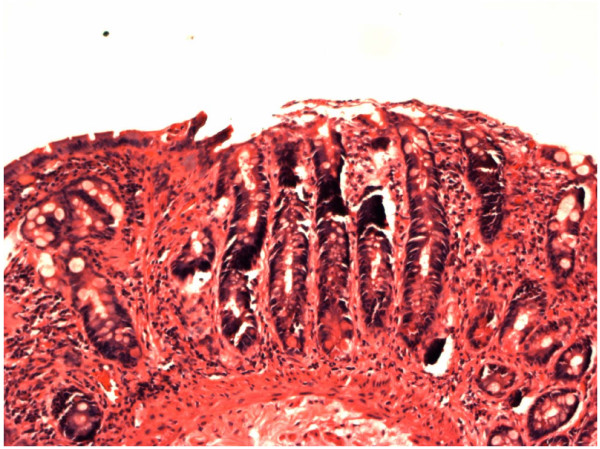
**Small-bowel biopsy sample of the patient who underwent positive seroconversion (Patient 4 in Table 1)**.

**Figure 2:**
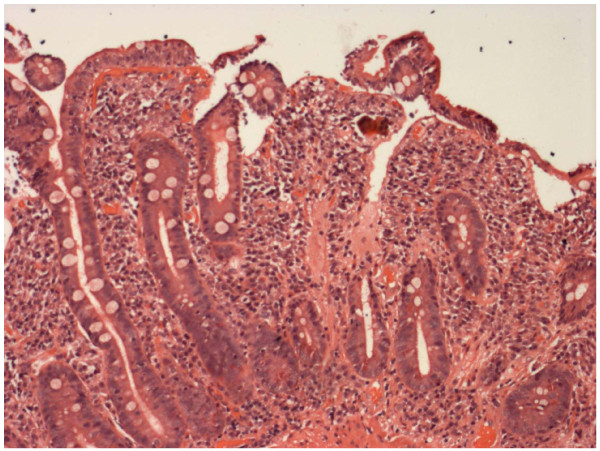
**Small-bowel biopsy sample of the patient who underwent positive seroconversion (Patient 5 in Table 1)**.

Of the 2216 individuals proving seronegative in the first examination, six had undergone positive seroconversion and five had biopsy-proven celiac disease (Marsh III); of these five new cases two reported minor abdominal complaints and three were asymptomatic. Thus, within three years, 0.23% developed celiac disease (CDb) and 0.24% underwent seroconversion (CDb+s). The values of IgA tTGA antibodies and EmA in the five patients with newly detected celiac disease are depicted in Table 1. The small bowel biopsy findings in patients with the lowest positive tTGA antibodies are shown in Figures 1 and 2. One of the five subjects (patient 4, Table 1) had immunosuppressive treatment (corticosteroids) upon the first and second screening. In 2005 the prevalence of celiac disease (CDb) was 2.34% in subjects aged 55 or more, and the frequency of biopsy-proven and seropositive individuals (CDb+s) 2.70% (Table 2).

**Table 1:** Serum Tissue Transglutaminase (tTGA) and Endomysial Antibody (EmA) Levels in the Five New Cases Who Underwent Positive Seroconversion and Were Found to Have Biopsy-Proven Celiac disease.

Gender, age (years), (by the time of diagnosis)	Screening in 2002		Screening in 2005	
	tTGA (Units)	EmA (titre)	tTGA (Units)	EmA (titre)
1: Male, 67	0.1	Not done	54.6	1:500
2: Female, 55	1.1	Not done	9.1	1:200
3: Male, 65	0.8	Not done	9.5	1:100
4: Female, 75	2.7	Not done	6.0	1:5^a^
5: Male, 66	0	Not done	7.1	0^b^

Reference Values for tTGA ≥ 5 Units and for EmA 1:≥5.

^a ^Small bowel mucosal villous morphology is shown in Figures 1 and ^b ^2

**Table 2:** Prevalence and Incidence of Celiac Disease (CD) and Seropositivity for IgA Class Tissue Transglutaminase (tTGA) and Endomysial (EmA) Antibodies in Patients Aged Over 55 Years.

Year, total population	Procedure	Number clinically detected	tTGA positive in screening	EmA positive in screening	Biopsy-proven cases in screening	Overall frequency of biopsy proven CD	Patients with CD and tTGA seropositive individuals
2002, 2815	Serum sampling						
2004, 2815	Recording of detected CD. First serologic analysis	25	48	43	35	60	69^a^
2005, 2216	Recording of detected CD. Second serologic screening	1	6	4	5^b^	6	7
Overall prevalence, N = 2815		0.92% (26/2815)	1.92% (54/2815)	1.70% (47/2815)	1.42% (40/2815)	2.34% (66/2815)	2.70% (76/2815)
95% confidence intervals		0.57–1.27%	1.41–2.43%	1.22–2.18%	0.98–1.86%	1.78–2.90%	2.10–3.30%

^a ^Four seropositive were found to have celiac disease between 2002–2004

^b^The incidence of celiac disease 2002–2005 was 0.23% (5/2216)

Of the 199 who underwent serologic screening for the first time in 2005, five had positive IgA tTGA antibodies and four positive EmA; three had villous atrophy compatible with celiac disease; biopsy was not possible in one who had moved away, and another subject declined due to serious heart disease. Thus the frequency of celiac disease was 1.5% (3/199), and when seropositives were included, 2.5% (5/199).

## Discussion

We have previously shown that the prevalence of celiac disease was higher in the elderly than what has been reported in the Finnish population among adolescents (1.5%)[7] or adults (2.0%) [8]. This difference might be due to diagnostic delay, which would increase the prevalence of the disease by time; the mortality of patients is low and comparable to that in the general population in Finland [9]. Nevertheless, from 2002 to 2005, the prevalence of biopsy-proven celiac disease (CDb) in this age group increased from 2.13% to 2.34%, and the combined prevalence of disease and seropositivity (CDb+s) from 2.45% to 2.70%. Such a combination makes sense: seropositivity for these specific antibodies in the absence of villous atrophy often indicates early developing celiac disease [10], and seropositive without villous atrophy may even benefit of dietary treatment [11].

There was a significant increase in the tTGA values in the five subjects who underwent seroconversion and were subsequently found to have biopsy proven coeliac disease (Table 1). This implies that there occurred a true seroconversion, though we did not have the opportunity to test again the original sera. It was also notable that the biopsy showed unequivocal villous atrophy and crypt hyperplasia even in the two patients with the lowest positive tTGA levels, shown in Figure 1 and 2.

It is not excluded that some of the five patients had had seronegative celiac disease and became seropositive later. On the other hand, there is some evidence that elderly people with newly detected celiac disease rather become seronegative by time [12]. This also means that the true frequency of celiac disease may be even higher than reported here.

Murray et al. [13] found that the incidence rates of celiac disease increased with age. A low index of suspicion by a physician may lead to diagnostic delay in recognition or to a distraction to other disorders. Apart from better diagnostics, a true increase in incidence may also occur [14]. We showed for the first time that the frequency of celiac disease was indeed increasing in elderly people, where clinically detected cases were recorded, and serologic screening has been carried out twice. The increase was thus not due to better diagnostics. Finland is considered genetically homogeneous, and there is no reason to believe that the frequency of celiac disease would be higher in Lahti region than in Finland in general.

The incidence of 0.23% during the study period implies that celiac disease may develop even at an advanced age. This again would imply that serologic testing should be repeated. Admittedly, the number of new cases was to low for any far-reaching conclusions. On the other hand, the annual incidence of about 0.1% indicates that the number of new cases may be 1% in 10 years. This percentage has in fact been achieved in many population screening studies. We would further emphasize that this incidence figure has been found in the general population with no suspicion of celiac disease, and with originally a high number of detected cases. The frequency of detected celiac disease in our general population is as high as 0.45% [15]. It is subject for further studies to establish whether the prevalence and incidence figures for celiac disease are even higher in elderly people belonging to the risk groups for the disease. For comparison, in relatives with celiac disease the incidence of new cases has been 1.7–4.5% within 7–12 years [16-18].

In those 199 screened for the first time, the prevalence of biopsy-proven celiac disease (CDb) was 1.5% (3/199), and when tTGA seroposives are included (CDb+s), 2.5%. These percentages are comparable to those detected in the main prospective study, supporting its results.

Earlier studies indicate that undiagnosed celiac disease may generate significant problems in the elderly. Freeman observed in his series of 30 celiac disease patients diagnosed over age 60 that they had suffered from many symptoms and had altogether 14 malignant conditions [19]. Hankey and Holmes [20] showed the diagnostic delay in the elderly to be considerable: 15 out the 35 aged 60 years or over had attended physicians for an average of 28 years with different complaints before the diagnosis. Their patients evinced good compliance with a gluten-free diet, and subsequently a significant improvement in their symptoms and signs. Similarly, Lurie et al. [21] found a significant lack in diagnosis, and a varied spectrum of manifestations in celiac patients diagnosed after the age of 60.

## Conclusion

In conclusion, the prevalence of celiac disease proved to be high in elderly people. Increased alertness and the free employment of serologic screening tests are warranted. One seronegative test result does not exclude forthcoming celiac disease. Our serial screening in the same population indicated that seropositivity and the disease may also appear later in life. This should be taken into account when considering celiac disease case finding and screening studies.

## Competing interests

The authors declare that they have no competing interests.

## Authors' contributions

AV participated in the study design, carried out the clinical studies, statistical analysis and drafted the manuscript. KK, MM and PC participated in the original study design and planning of the protocol, in the analysis of the data, and in writing and revising the manuscript. IK and HP carried out the endoscopy examinations and participated in the clinical examination of the patients. LL participated in the study planning, study protocol and drafted the manuscript. RV collected the data and sera, and participated in the study design. All authors have read and approved the final manuscript.

## Pre-publication history

The pre-publication history for this paper can be accessed here:

http://www.biomedcentral.com/1471-230X/9/49/prepub
